# Unpicking the Gordian knot: a systems approach to traumatic brain injury care in low-income and middle-income countries

**DOI:** 10.1136/bmjgh-2018-000768

**Published:** 2018-03-25

**Authors:** Tom Bashford, P John Clarkson, David K Menon, Peter J A Hutchinson

**Affiliations:** 1 NIHR Global Health Research Group for Neurotrauma, University of Cambridge, Cambridge, UK; 2 Engineering Design Centre, Department of Engineering, University of Cambridge, Cambridge, UK; 3 Division of Anaesthesia, Department of Medicine, University of Cambridge, Cambridge, UK; 4 Division of Neurosurgery, Department of Clinical Neurosciences, University of Cambridge, Cambridge, UK

**Keywords:** health systems, traumatology, neurology, health services research, surgery

**Summary box**

- Traumatic brain injury is a major global health issue and requires a functional health system to be optimally managed—often lacking in those low-income and middle-income countries where the burden of this disease is highest.
- Clinical care is the emergent property of a complex, adaptive, sociotechnical system: generating improvements in care can benefit from a systems approach.
- Systems Engineering can inform a systems approach to healthcare improvement, complementing more traditional improvement techniques.
- A systems approach may be a valuable tool in trying to understand, and improve, the clinical care of patients with traumatic brain injury in low-income and middle-income settings.

The Global Burden of Diseases, Injuries and Risk Factors Study showed that in 2010 trauma accounted for 9% of the world’s deaths—around 5 million people—while also resulting in millions of non-fatal injuries with resultant disability. Around 90% of injury-related deaths occurred in low-income and middle-income countries (LMICs), which also saw the greatest rise in these injuries due to road traffic collisions.^1^ More recent global health estimates from WHO for 2015 show a similar picture.^2^ As a disease subtype, traumatic brain injury (TBI) is one of the most devastating, with clinical, societal and economic sequelae.^3^ It is also startlingly common with an estimated 50 million or more cases per year; enough for half of the world’s population to suffer a TBI in their lifetime and again disproportionately affecting lower-income regions.^4^

TBI is a heterogeneous condition, which can be difficult to both manage and prognosticate in even the best resourced settings and involves an array of prehospital, acute treatment and rehabilitation services.^4 5^ These are interdependent meaning improvement in any one area of care may not be reflected in overall clinical outcome. While aggressive treatment of TBI can minimise disability in many patients, prevention of mortality can also result in survival with severe disability in others, making the decision to intervene or not an ethically complex one, since the health systems resources required to effectively manage TBI are significant.^6^

An additional layer of complexity is added by the wider LMIC environment: fragile health systems, a lack of coordinated social support and inadequate infrastructure complicate the management of TBI in both the acute and the rehabilitative phase. Applying the current clinical evidence base, largely generated in high-income countries, may not be appropriate in LMICs. Ethical considerations will be culturally and contextually dependent, and measures of outcome may be hard to agree on.^7^ Health systems strengthening is recognised as a key challenge in LMICs, yet relating wider systems strengthening to the care of a specific, although heterogeneous, clinical entity is a particular challenge.^8 9^

What is required to overcome these myriad challenges? Better epidemiological data are essential, both to characterise and quantify injuries being sustained in different contexts, and to map their current management. Clinical medicine clearly has a role to play, with guidelines, protocols and pragmatic trials based in LMICs needed to generate an appropriate and applicable evidence base to guide management, alongside the development of innovative technologies appropriate to the LMIC context.^7^ However, these will only solve part of the problem. Allied disciplines such as Improvement Science, Implementation Science, Operational Research and Human Factors Engineering may all offer insights as to not only *what* to do but *how* to do it.^10–14^

A central tenet of many of these disciplines is the concept of medical care as delivered by a system. Systems thinking in healthcare is endorsed by WHO as particularly apposite for improvement in healthcare in LMICs, while in the field of global surgery, the *Lancet Commission on Global Surgery 2015* notes the importance of focussing on systems with regard to surgical improvement.^9 15 16^ More specifically in TBI, the importance of the health system is highlighted in the recent *Lancet Neurology Commission on Traumatic Brain Injury*.^4^ However, a universal definition of systems thinking, or even of a system in the context of healthcare, is lacking. There is an increasing recognition that healthcare represents a *complex, adaptive, sociotechnical system* consisting of people and equipment, processes and institutions. The complexity arises from both the number of, and the variability in, the interactions between these different components. Indeed, several elements of complex systems may be observable in healthcare including emergence, path dependence, self-organisation, non-linearity and non-scalability.^8 17–19^

TBI care may be seen then as the apotheosis of a systems problem: a complex, heterogeneous, clinical entity which requires a functional macro-healthcare system and a coordinated micro-clinical system, has variable and controversial measures of outcome and has a significant cultural and ethical component to its management. The development of a systems understanding of TBI care appears essential to coordinate the more traditional approaches to healthcare improvement.

Developing a systems approach to understanding and improving TBI care is operationally challenging. The disciplines listed above may rely on a systems understanding but rarely provide examples as to how to model complex systems so that they can be rationally re-engineered. Furthermore, there is little to guide researchers as to how to contextualise the micro-system of clinical care into the macro-system of national health service delivery to ensure these are mutually supportive of each other. Recently, the use of established dynamic systems modelling techniques such as Systems Dynamics Modelling (SDM) and Agent Based Modelling (ABM), in addition to static techniques such as network analysis and scenario planning, have been proposed as helpful to understanding healthcare improvement in LMICs.^20^ In fact, an array of techniques for modelling healthcare have been put forward, and work done to understand the place of each in intervention design.^21–23^ In addition, there has been a growth in interest in techniques borrowed from industry such as Lean and Six Sigma.^24^

**Figure 1:**
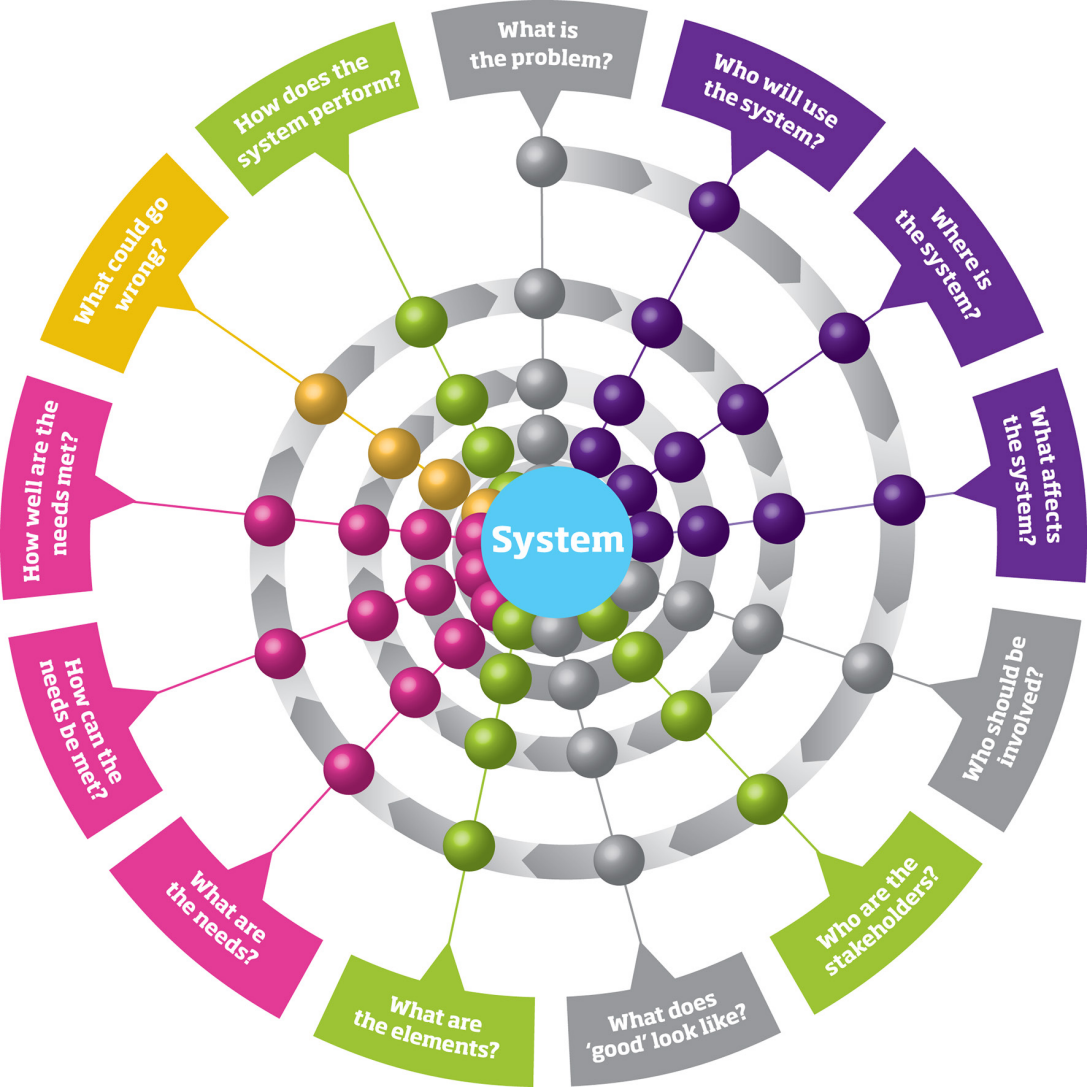
A systems approach as an ordered series of questions regarding general improvement (grey), people (purple), systems (green), design (pink) and risk (orange). Reproduced with permission from Engineering Better Care, Royal Academy of Engineering, Academy of Medical Sciences and Royal College of Physicians, 2017

In this arena, the experience of the Engineering community may offer crucial insights.^25^ This has recently been highlighted through a joint project by the UK Royal Academy of Engineering, the Royal College of Physicians and the Academy of Medical Sciences.^26^ Their report, ‘*Engineering better care: a systems approach to health and care design and continuous improvement*’, outlines the value that an engineering-informed systems approach can bring to healthcare, through focussing on understanding the people, systems, design and risk involved when pursuing healthcare improvements. This approach can be summarised in an iterative series of questions, which need to be answered as a project progresses (figure 1). The application of Systems Engineering to patient safety has been widely adopted, but the Royal Academy report emphasises the role a broader systems approach can play in healthcare improvement, with Systems Engineering complementing existing healthcare improvement tools.^27–30^ Crucially, such a systems approach brings together many of the discrete techniques such as SDM and ABM, but includes a wide variety of other quantitative and qualitative methodologies.

How do these four tenets of people, systems, design and risk inform our understanding of complex systems? The first insight is that systems can be interrogated through the lens of many different stakeholders, and that a full understanding of the system can only come about through rigorous and extensive engagement with these stakeholders. The second is that systems can be represented in a variety of ways, and that matching the right model to the right problem is fundamental to generating a common understanding and possible solutions. Third, interventions to improve care benefit from a design process of iterative improvement based on this understanding of people and systems, and fourth that all interventions create risk—both of unintended consequences and of wasted endeavour—which can be formally modelled. Crucially, such a systems approach does not seek to generate generalisable answers to healthcare problems; rather it seeks to use a generalisable process to design context-dependent solutions. It is this context dependence that suits it so well to address some of the difficulties that arrive with the improvement of TBI care in LMICs.

**Figure 2:**
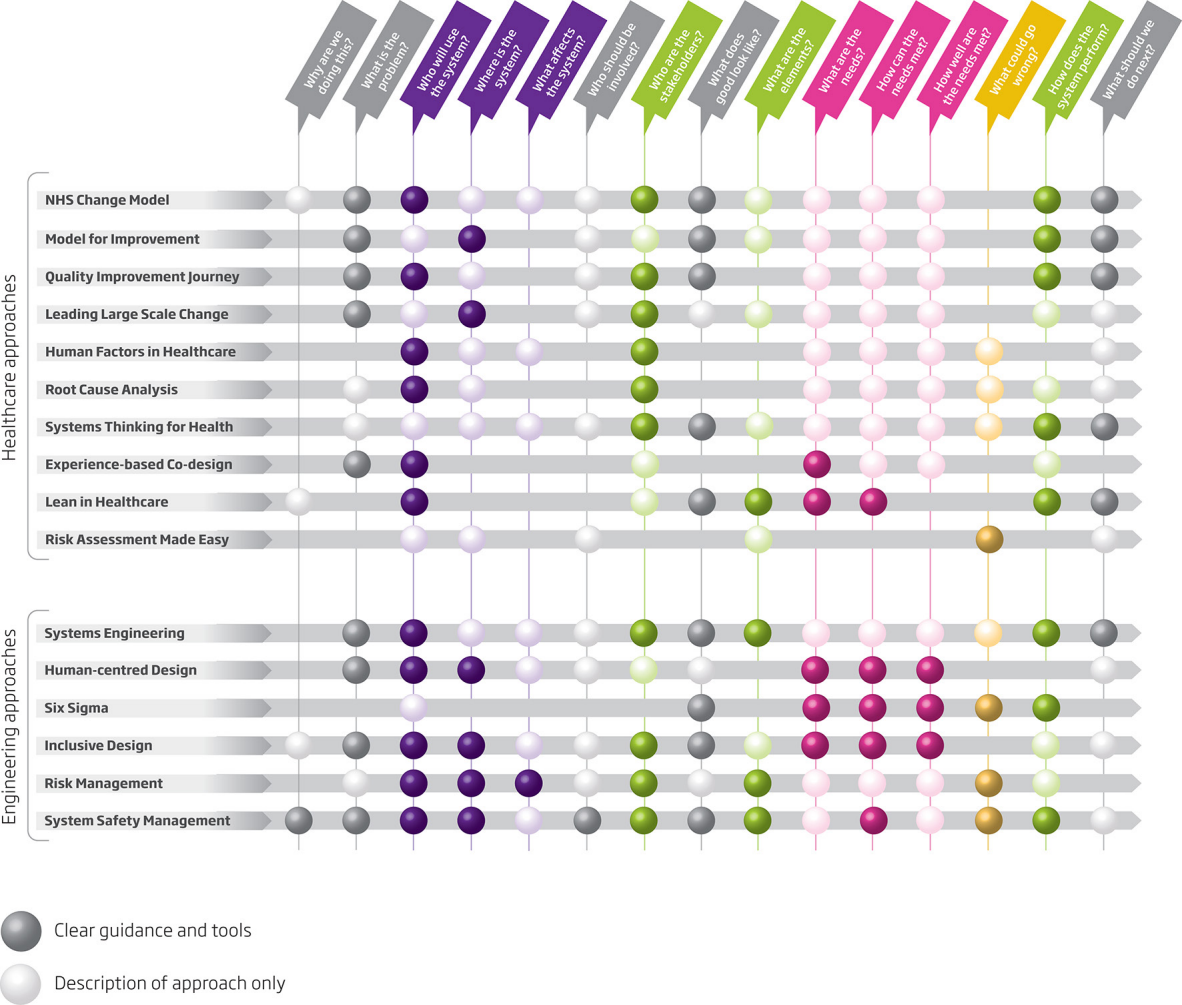
The ability of both engineering and traditional approaches to healthcare improvement to answer the key questions of a systems approach. Reproduced with permission from Engineering Better Care, Royal Academy of Engineering, Academy of Medical Sciences and Royal College of Physicians,2017

What does a systems approach offer alongside existing schools of improvement or implementation? Figure 2 shows how the series of questions proposed as key to solving systems problems may be answered by both traditional and engineering-informed approaches. While traditional approaches reference many of the questions, they often fail to provide a methodological basis for answering them. In addition, an engineering-informed systems approach provides an academic and operational basis to bridge the gap between the various levels of the healthcare delivery system.

Arguably, the most important element to the LMIC context is that many of the systems modelling techniques used by engineers do not require high-resolution data as a prerequisite. Many of these modelling techniques are based around narrative or workshop data, and use this to graphically represent problems to allow consensus problem solving.^19 31^ While quantitative modelling and simulation can augment this process, they are not essential.^19^ Mixed-methods approaches are ideal in LMICs where robust data can be hard to obtain; a ‘soft-systems methodology’ used as part of a systems approach provides a focused, proven method of pragmatic problem structuring with the available data.^32^ On a more philosophical level, such a systems approach recognises that health systems are best interrogated through an understanding of stakeholders’ perspectives, and that systems design starts first with the elucidation of those stakeholders’ needs. This has clear cross-over with many of the principles of partnership and codevelopment, which have grown out of the global health community.^33–38^

How can we relate this systems approach back to the clinical problem of TBI in LMICs? Consider the four themes or people, systems, risk and design. The stakeholders are varied and may include neurosurgeons, anaesthetists, patients, nurses, administrators, non-governmental organisations, national and international societies, funding agencies, academics and religious leaders. The systems involved include international, national, regional and local institutions, and span prehospital, acute and rehabilitative care. There is a risk of wasted endeavour, of active harm or of failing to monitor genuine improvement. Any proposed change to care must therefore be carefully designed in order to explicitly account for these elements. A given intervention—such as guideline development, equipment provision or clinical training of neurosurgeons—may cause a number of possible changes to this complex picture. The better our understanding of this system before an intervention is planned, the better our ability to improve outcomes for patients with TBI both directly and indirectly. In addition, by understanding the system there is the possibility for synergy with other improvement efforts within the healthcare ecosystem.

Critics of the systems approach advocated here argue that many of its principles are ‘common sense’; however, the realisation of the Engineering community is that common sense is rarely common and that a framework to embed it in both operational and research work has enormous value. Systems Engineering has an impressive pedigree outside of the medical field and is widely used in other safety-critical industries such as aviation, spaceflight, defence and manufacturing.^39–44^ The tools and techniques it has developed provide powerful insights into the planning and implementation of interventions to complex systems, and incorporating these into a wider systems approach may be of enormous benefit to clinicians and academics seeking to address global health challenges.
